# The new Regulation on clinical trials in relation to radiopharmaceuticals: when and how will it be implemented?

**DOI:** 10.1186/s41181-019-0055-6

**Published:** 2019-01-11

**Authors:** Iván Peñuelas, Daniëlle J. Vugts, Clemens Decristoforo, Philip H. Elsinga

**Affiliations:** 1 Radiopharmacy Unit, Department of Nuclear Medicine, Clínica Universidad de Navarra (UNAV), IdiSNA, Pamplona, Spain; 2 Department of Radiology & Nuclear Medicine, Amsterdam Neuroscience, VU University Medical Center, Amsterdam, the Netherlands; 3 Department of Nuclear Medicine, Medical University of Innsbruck, Innsbruck, Austria; 4 Department of Nuclear Medicine and Molecular Imaging, University of Groningen, University Medical Center, Groningen, the Netherlands

The development of radiopharmaceuticals with high specificity and high potential is increasing dramatically. This opens new doors in clinical research and changes clinical management resulting in an increased pressure to conduct controlled clinical trials. The nuclear medicine community is currently facing challenges during the transition phase of the clinical trial regulations.

In the EU the legislation related to clinical trials will be ruled by Regulation 536/2014, the so-called “Clinical Trial Regulation”. There are substantial changes when we compare this new regulation with the old (albeit currently applicable) Directive 2001/20.

## From the directive to the regulation: Need for a change and key benefits

Until 2014, the Directive and its transposition to the national legislation in the different EU member states has resulted in many legal variations within Europe, great obstacles in the execution of clinical trials, the imposition of highly demanding and expensive regulatory requirements (irrespective of the level of risk), a substantial decrease in investigator (rather than drug company) driven studies and an extremely slow pace for implementation of trials in practice.

Even the European Commission stated that the old Directive needed to be replaced for 3 main reasons (Q&A: New rules for clinical trials conducted in the EU 2014): (1) the criticism by patients, researchers and industry alike for its disproportionate regulatory requirements; (2) the high costs and a lack of harmonization of the applicable rules necessary for multinational clinical trials and (3) the significant decline in the number of clinical trials in the EU to which the restrictions imposed by the Directive had contributed to.

Therefore, the new Regulation was aimed to restore the EU’s competitiveness in clinical research, foster the development of new and innovative treatments and medicines and bring patient-oriented research back to Europe. The general changes introduced by the Regulation have been reviewed elsewhere (Tenti et al. 2018). In summary, the Regulation establishes a harmonized electronic submission and assessment process for clinical trials conducted in multiple Member States, favors collaboration, information-sharing and decision-making between and within Member States, introduces increased transparency of information on clinical trials and fixes highest standards of safety for all participants in EU clinical trials (Clinical Trial Regulation 2018).

As a whole, the Regulation is substantially focused on patient safety and reasonable and proportionate risk assessment, simplifying approval procedures that will also facilitate multicenter trans-national clinical trials with the creation of the centralised EU submission Portal. The new and more efficient procedures will ensure first of all patient safety and public health and will be designed to avoid administrative delays implementing the prompt answer for clinical trial applications but ensuring also strict scientific and ethical reviews.

The Regulation also takes much better into account the real risk for the subjects involved in a clinical trial by adapting the regulatoryion burden in relation to the risk posed.

## Entry into to force and applicability of the new regulation

It is worth mentioning that due to the complex timelines associated with the Regulation, it is not already applicable although it came already into force back in June 2014. Unfortunately, there is not a fixed date for its applicability, but it will happen 6 months after the European Commission will publish a note confirming the full functionality of a new EU Portal and Database once it has undergone an independent audit. The EU Portal and Database, that is being developed by the European Medicines Agency (EMA), will be the backbone of the new regime for clinical trials in Europe. It will facilitate and harmonize the application for clinical trials authorisation, in particular in case of international multicentre clinical trials. It will facilitate the assessment carried out by regulatory authorities and the access to clinical trials information by the general public.

The system will be a single entry point for submitting clinical trials in the EU and provide a database, which will contain collaboration tools, workflow and document management capabilities, accessible via individual workspaces for the sponsor, the authorities and the public.

The EU Portal and Database initial delivery timeframe was fixed for December 2015, but it has been constantly postponed due to technical difficulties faced in the development of the IT systems. Currently, according to EMA’s website, the most probable time frame for the applicability of the Clinical Trial Regulation is late 2020.

However, once in force, there will be a transition period in which both the old and the new legislation on clinical trials will coexist. Within this period there is the choice of submitting within the old or the new system and trials running in the old system can run for a maximum of 3 years after implementation of the new regulation.

In the end, it will have taken almost 20 years (2001–2020/23) to change crucial legislation for the conduct of research involving human subjects and hence for the development of better healthcare for our patients. We would definitely need the implementation of much faster and efficient procedures in the EU to react on the constant developments in drug development in general and radiopharmaceuticals in particular.

## Radiopharmaceuticals in the new regulation

It is important to consider the differences established by the Regulation between therapeutic and (some) diagnostic radiopharmaceuticals. Therapeutic radiopharmaceuticals have no special treatment at all in the new regulation (as was the case for all radiopharmaceuticals in the old Clinical Trial Directive) and are considered in every aspect in the same way as any other medicinal product used in a CT.

However, the new Regulation introduces substantial changes in the field of diagnostic radiopharmaceuticals: *“preparation of radiopharmaceuticals used as diagnostic investigational medicinal products where this process is carried out in hospitals, health centers or clinics, by pharmacists or other persons legally authorized in the Member State concerned to carry out such process, and if the investigational medicinal products are intended to be used exclusively in hospitals, health centers or clinics taking part in the same clinical trial in the same Member State”* (Art. 61.5.b of Regulation 536/2014).

These changes are:

1. **No need for having the authorization for manufacturing** and import of investigational medicinal products is referred to in art. 61.1 of the regulation which makes it much easier to prepare diagnostic radiopharmaceuticals when used as Investigation Medicinal Products (IMPs) in clinical trials.

While only diagnostic RPs are covered by the exemption from manufacturing authorization by art. 61.5.b, specific therapeutic RPs could be considered as magistral or officinal preparations according to the national legislation of the corresponding EU country. In this case, the exception 61.5.c would be applicable, which specifically refers to “medicinal products referred to in points (1) and (2) of Article 3 of Directive 2001/83/EC” (this paragraph defines magistral and officinal preparations). In addition, the text talks about “preparation”, and not “manufacturing” (for a discussion on this topic, see Decristoforo & Patt, 2017) but only IMPs (Investigational Medicinal Products) are included in the “exception” of art. 61.5.b, while nothing is said about NIMPs (non-investigational medicinal products) and AMPs (auxiliary medicinal products). In many cases in-house prepared RP might be considered as NIMPs or AMPs in a Clinical Trial. Finally, art. 61.5.b does not exclude the use of prepared IMPs in a different centre than in which it was prepared (whenever it is a hospital, health centre or clinic) with the only condition that it will be in the same member state.

2. No need for GMP production of the RP included in the exception of art 61.(5)

As a consequence of Regulation 536/2014 two new legislation documents in relation to GMP have been released: (1) Directive 2017/1572, the new GMP Directive that repeals the old GMP Directive 2003/94, and (2) Regulation 2017/1569, a new GMP Regulation for IMPs (that does not apply to those RPs included in art. 61(5) of Regulation 536/2014). They will become effective with the implementation of the new regulation and provide a clear context in relation to those IMPs covered by the regulation.

However, a common framework for the preparation of the RP considered in the exclusion of art. 61(5) of the Clinical Trial Regulation is still lacking. The Regulation itself says in its preamble that “*Member States should make those processes subject to appropriate and proportionate requirements to ensure subject safety and reliability and robustness of the data generated in the clinical trial”.* Therefore, in order to prevent further lack of harmonization, it remains an important task for our Nuclear Medicine community in the future to provide a clear position and guidance on this topic, e.g. in the framework of EANM to support the community with a common understanding the extent of the quality framework for diagnostic IMPs being affected by the exclusion of art.61(5).

3. Simplified labelling of diagnostic RP used as IMPs or AMPs

Articles 66 and 67 of Regulation 536/2014 refer to labelling of unauthorized investigational and unauthorized auxiliary medicinal products and labelling of authorized investigational and authorized auxiliary medicinal products, respectively. Art. 68 of the Regulation states that articles 66 and 67 shall not apply to radiopharmaceuticals used as diagnostic IMPs or as diagnostic AMPs, and hence a simplified labelling of this type of RPs is possible. While not being such a relevant change as the ones mentioned in points 1 and 2 (related with authorization and GMP production), this exception will in practice facilitate the use of adequate labels to the specific characteristics of the RPs, in particular, related to radiation safety considerations.

As stated in the introduction, legislation on clinical trials is in a transition phase. The new Regulation 536/2014 provides new opportunities in particular harmonizing multinational approaches and the delay in its implementation has caused great uncertainty in planning new studies in which we go from “bench to bedside”. The regulation for the first time recognizes radiopharmaceuticals to be special which is a big step forward as compared to the current legislation. Harmonization within Europe also bears the risk that authorities will take fewer responsibilities and will be compared in a greater “European Concert”, and national “specialities” will become less acceptable. It also should be stated that the regulation does not reduce the type of documentation required for submission, and therefore additional guidance on requirements for new radiopharmaceuticals have to complement this process. A recent step in this direction is a recent draft guideline from EMA on “non-clinical” requirements for radiopharmaceuticals (EMA/CHMP/SWP/686140/2018 2018).

The Nuclear Medicine Community cannot rely on the changes that are already underway but constantly has to point out and promote the speciality of our field also within the regulatory framework ensuring a risk proportionate approach to ensure the progress towards novel medicines for patients also in the coming years. Especially for RPs with their low-risk profile compared to many other drugs, a clear strategy and guidance is needed to allow professionals to concentrate efforts towards the real challenges in the provisions of safe and efficacious RPs. Practical examples and data should be provided by the community involving authorities and decision-makers and must be made available to facilitate objective and unbiased development of risk-based approaches.
